# A simple non-perturbing cell migration assay insensitive to proliferation effects

**DOI:** 10.1038/srep31694

**Published:** 2016-08-18

**Authors:** Honor L. Glenn, Jacob Messner, Deirdre R. Meldrum

**Affiliations:** 1 Center for Biosignatures Discovery Automation, The Biodesign Institute, Arizona State University, Tempe, AZ 85287 USA

## Abstract

Migration is a fundamental cellular behavior that plays an indispensable role in development and homeostasis, but can also contribute to pathology such as cancer metastasis. Due to its relevance to many aspects of human health, the ability to accurately measure cell migration is of broad interest, and numerous approaches have been developed. One of the most commonly employed approaches, because of its simplicity and throughput, is the exclusion zone assay in which cells are allowed to migrate into an initially cell-free region. A major drawback of this assay is that it relies on simply counting cells in the exclusion zone and therefore cannot distinguish the effects of proliferation from migration. We report here a simple modification to the exclusion zone migration assay that exclusively measures cell migration and is not affected by proliferation. This approach makes use of a lineage-tracing vital stain that is retained through cell generations and effectively reads out migration relative to the original, parental cell population. This modification is simple, robust, non-perturbing, and inexpensive. We validate the method in a panel of cell lines under conditions that inhibit or promote migration and demonstrate its use in normal and cancer cell lines as well as primary cells.

Most mammalian cells have some capacity to move over or through an extracellular matrix. Cell migration plays a critical role in embryogenesis^1^, tissue remodeling, and wound healing^2^. However, misregulated cell migration can contribute to pathology as in autoimmune diseases^3^,^4^ and, most notably, metastatic cancer^5^,^6^. Because of the profound implications of cell motility for human health, there is long-standing interest in methods for measuring cell migration that are non-perturbing, rapid, inexpensive, robust, and high-throughput. One of the simplest and longest used methods is the scratch or wound-healing assay^7^. In this approach, cells are grown to confluence then the monolayer is mechanically scratched with an object such as a narrow pipette tip to form a denuded region. Motility is assessed by the rate at which cells fill in the opened area. This simple approach continues to be used despite a number of drawbacks. The size and edge features of the scratch are difficult to standardize, the properties of the migration surface are not well controlled since it can be modified by the cells that initially grew on it, and the removal process necessarily exposes the culture to dead and damaged cell components. These deficiencies are overcome in the barrier or exclusion zone assay^8^. In this approach, a portion of the cell growth surface is physically blocked during cell seeding and attachment such that a cell-free region is formed. Migration is initiated by removal of the blockade, then migration is evaluated as cells fill the newly exposed surface. Another popular migration assay is the Transwell or modified Boyden chamber assay^9^. In this assay, cells are seeded onto a membrane filter submerged in medium. Over time the cells migrate through pores to the underside of the membrane. At the assay endpoint, cells are removed by wiping from the top of the membrane, then the cells remaining on the bottom are stained and quantified by counting or other method. This method is best suited to lower throughput studies as it is usually done in a 24-well format and requires a labor intensive cell removal step. Recent technological advances have brought new approaches such as electrical impedance assays, microfluidic platforms, and cell tracking methods^10^,^11^. However, these newer approaches remain less popular due to increased complexity and the requirement for expensive equipment.

With the exception of time-lapse imaging with tracking of individual cells^12^, most migration assays are based on the simple premise of allowing cells to migrate then quantifying cells that are present in an area that was cell free at the start of the migration period. A significant drawback of these approaches is that in the majority of cases, cell migration is conflated with cell proliferation^11^. This problem is commonly ignored or dealt with by reporting results as “colony expansion”, recognizing that the final distribution of cells represents the combined contributions of movement and proliferation. Alternatively, attempts to achieve a pure migration response are made by inhibiting cellular proliferation during the assay^13^,^14^. Mathematical models have also been devised to distinguish migration from proliferation^15^.

We describe here a new method for quantifying cell migration that is essentially blind to the confounding influence of cell proliferation. This is accomplished by use of a fluorescent cell label that distributes to daughter cells with a constant dilution factor at each cell generation, thereby providing a read-out of migration that is normalized to the original cell population. We demonstrate application of the approach with cell lines and primary cells using a commercially available barrier assay. However, the method is easily extensible to any exclusion zone-type migration assay format.

## Results

### Use of lineage tracing dyes in an exclusion zone migration assay

**Figure 1:**
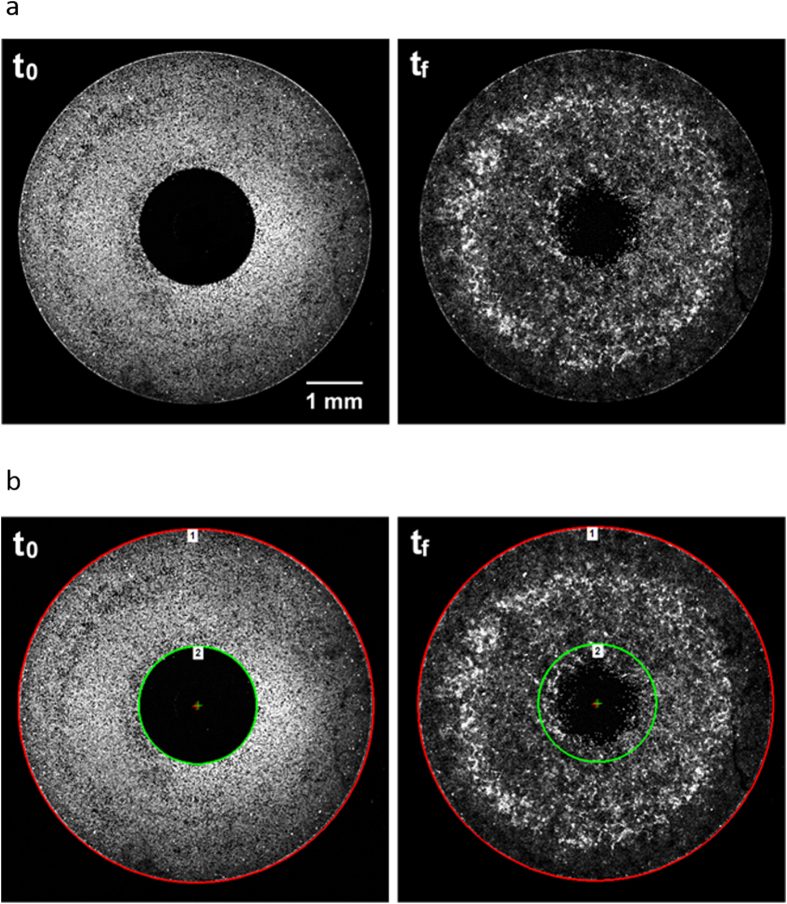
Image analysis method for using a lineage tracing dye to quantify cell migration. (**a**) CP-A cells in Oris™ migration wells were labeled with CellTrace Violet and the pre-migration, t_0_ image was captured (left panel). Cells were allowed to migrate for 14 hours, then the final image, t_f_, was captured (right panel). (**b**) Two regions of interest (ROIs) were created on the t_0_ image (left). ROI-1 (red) includes all cells, ROI-2 (green) delineate the edge of the cell-free exclusion zone. Both ROIs are pasted, maintaining their relative postions, onto the post-migration, t_f_, image (right). Migration is assessed as the percent of flourescent signal in ROI-2 relative to total fluorescence (ROI-1).

We employed a commercially available, live cell stain optimized for lineage tracing, in development of an improved cell migration assay. We combined this labeling approach with the Oris™ 96-well migration kit, an exclusion zone-type assay (Platypus Technologies, Madison, WI). Cells were seeded into a 96-well plate containing silicon inserts that form a circular, cell-free zone in the center of each well. After adhesion, the inserts were removed and the cells were stained with either 4 μM CellTrace Violet (CV) or 20 μM CellTrace Far Red (CFR) (Thermo Scientific, Rochester, NY). Optimization experiments identified these concentrations as the minimum amount of dye that produced a robust signal and reproducible results. The wells were immediately imaged for the t_0_, pre-migration time point. Cells were allowed to migrate for 12–24 hours depending on cell type, then imaged again for the final time point, t_f_ (Fig. 1a). The t_0_ image was then used to create 2 circular regions of interest (ROIs): ROI-1 that encompassed all the cells, by circumscribing the inner diameter of the well and ROI-2 that delineated the cell-free exclusion zone (Fig. 1b). These ROIs were then pasted together, maintaining their relative orientation, onto the t_f_ image and centered by alignment to the well periphery (Fig. 1b). The total intensity within each ROI was recorded and the percent cell migration was calculated from the amount of signal that was detected in the exclusion zone as: relative migration = (I_ROI-2_/I_ROI-1_)*100, were I is the sum of intensity within the given ROI.

### Effects of lineage tracing dye on cell migration and proliferation

**Figure 2:**
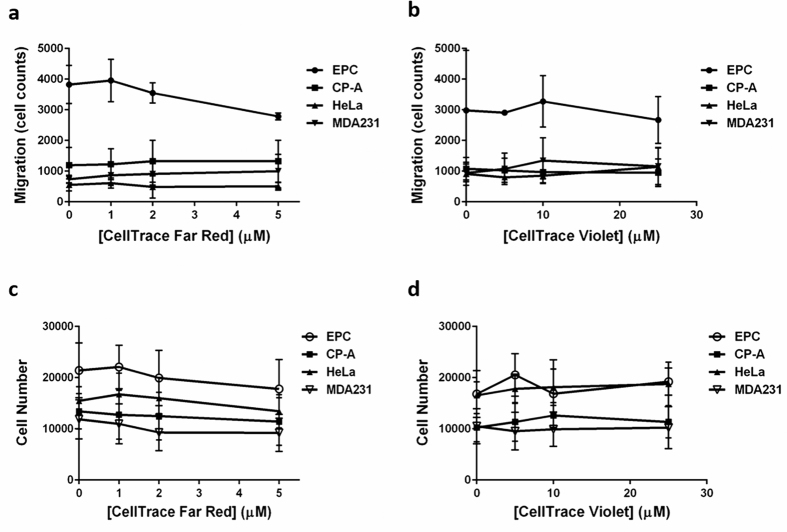
CellTrace dyes do not alter cell migration or proliferation. (**a**) EPC2 (EPC), CP-A, HeLa, and MDA-MB-231 (MDA231) cells were seeded into migration wells and labeled with various concentrations of CFR before migration. After migration, cells were labeled with the nucleic acid stain SYTO 24 and imaged. NIS-Elements was used to quantify the number of nuclei in the exclusion zone. Data were pooled from two independent experiments each consisting of four replicates. CFR treatment had no discernible effect on migration (p = 0.99). (**b**) Similar to (**a**) except cells were labeled with the indicated concentrations of CV during migration. This treatment had no discernible effect on migration (p = 0.87) (**c**) Cells were seeded subconfluently into a 96-well plated without migration barriers. Cells were exposed to CFR at the indicated concentrations for 24 hours. Then cells were labeled with SYTO 24 and total cells were enumerated using NIS-Elements. CFR had no discernible effect on cell numbers after 24 hours (p = 0.96). (**d**) Similar to (**c**) except cells were exposed to the indicated concentrations of CV. This treatment had no measurable effect on cell numbers (p = 0.80). Error bars indicate SEM. Data were analyzed by two-way ANOVA with Dunnett’s multiple comparisons test.

We first sought to determine whether the fluorescent stain would have any impact on relevant cellular processes, such as proliferation or cell motility. These parameters were tested in four human, epithelial cell lines: EPC2, derived from normal, esophageal, squamous epithelium; CP-A, derived from non-dysplastic, Barrett’s esophagus epithelium; HeLa, derived from cervical adenocarcinoma; and MDA-MB-231, derived from metastatic breast adenocarcinoma. The effects of the CellTrace dyes on cell migration were evaluated using the Platypus migration assay according to the manufacturer’s protocol. Cells were allowed to migrate in the presence of various concentrations of CV or CFR. None of the cell lines were affected at the dye concentrations tested (Fig. 2a,b). P-values were 0.87 and 0.99 for CFR and CV, respectively. At 5 μM, the highest concentration of CFR, there was a small decrease in the mean migration values. Though it did not meet the criterion for significance, in subsequent experiments we used a maximum concentration of 4 μM of this label. It should be noted that this assay is based on a simple exclusion zone filling principle and does not discriminate between contributions from cell migration and cell proliferation. Therefore, we also tested independently for any effect on cell proliferation. We found that cell density did not differ from controls 24 hours after exposure to either dye in any of the cell lines (p = 0.80 for CFR and 0.96 for CV) (Fig. 2c,d).

### Retention of dyes in adherent cells

**Figure 3:**
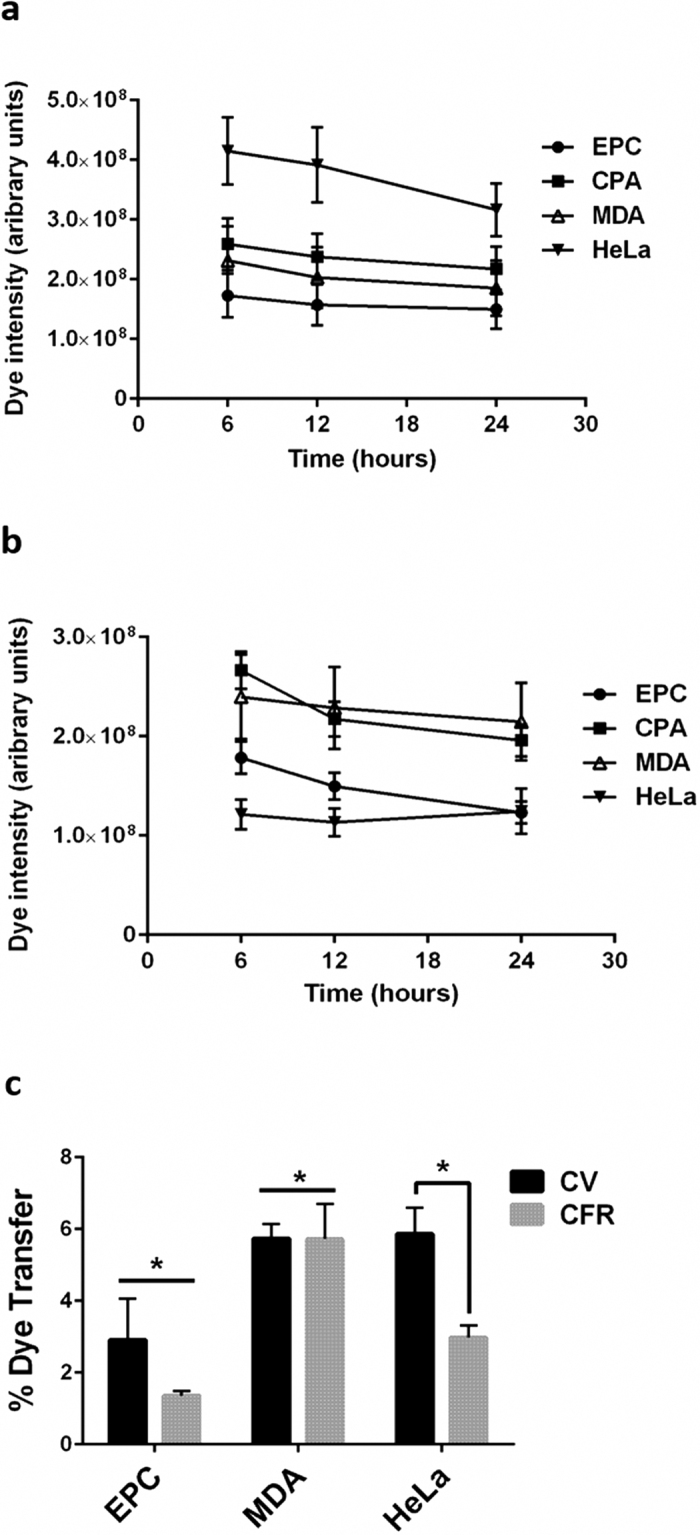
CellTrace dye retention. Adherent EPC2 (EPC), CP-A (CPA), MDA-MB-231 (MDA), and Hela cells were labeled with CellTrace Violet (**a**) or CellTrace Far Red (**b**). Cells from the entire well were imaged at 6, 12, and 24 hours post staining. There was no significant change in CellTrace Violet signal intensity in any cell type. There was a significant loss in CellTrace Far Red in EPC2 and CP-A cells at 24 hours (p = 0.03) but not at 12 hours. Data were pooled from two independent experiments each conducted in duplicate. (**c**) Cells were labeled with CellTrace Violet or CellTrace Far Red in suspension, then cells were seeded as single-stained populations or mixed samples and imaged 24 hours post-seeding. Image cytometry was conducted on images of 300–500 cells. Dye transfer is calculated as the intensity of the transferred dye signal relative to the intensity of the same signal in the single-labeled population. Data were pooled from two independent experiments each conducted in triplicate. Error bars indicate SEM, results were analyzed by one-way ANOVA. * indicates p < 0.05.

CellTrace dyes readily permeate the plasma membrane and bind to intracellular proteins. They have been shown to be stably retained in the cytosol and have minimal transfer from cell to cell, except from parent to daughter cells^16^,^17^. However, most of these analyses have been done on cells in suspension via flow cytometry. Therefore we evaluated how well the dyes are retained in adherent cells under cell culture conditions used during migration assays. We labeled adherent cultures of EPC2, CP-A, MDA-MB-231, and HeLa cells then imaged them at 6, 12, and 24 hours after labeling. We found that CV was very well retained in all cell type and there was no significant loss of signal up to 24 hours (Fig. 3a), the maximum migration time. CFR was also well retained, with no loss of signal in MDA-MB-231 or and HeLa cells. However there was a small, measureable decrease in signal from EPC2 and CP-A cells at 24 hours (p = 0.03) with this dye (Fig. 3b). CFR signal was not reduced at 12 hours for any cell type.

We also measured the degree of dye transfer between cells in high density culture. Cells labeled with CV or CFR were seeded either separately or in mixed culture for 24 hours, then imaged. Dye transfer in the mixed cultures was calculated as the increase in blue signal in CFR-labeled cells and the increase in far red signal in the CV-labeled cells relative to background signal measured in the single-labeled cultures (Fig. 3c and Supplementary information, Figure S1). Both dyes were well retained in the respectively labeled population; overall, CV transferred at less than 5% and CFR at less than 4%. There was some cell type dependent variability in this measure of dye retention. EPC2 cells had significantly lower dye transfer and MDA-MB-231 had significantly higher transfer than the other cell lines (p < 0.05). HeLa cells had a differential response to the two labels, retaining CFR more effectively than CV (p < 0.05).

### Stimulated and inhibited cell migration

**Figure 4:**
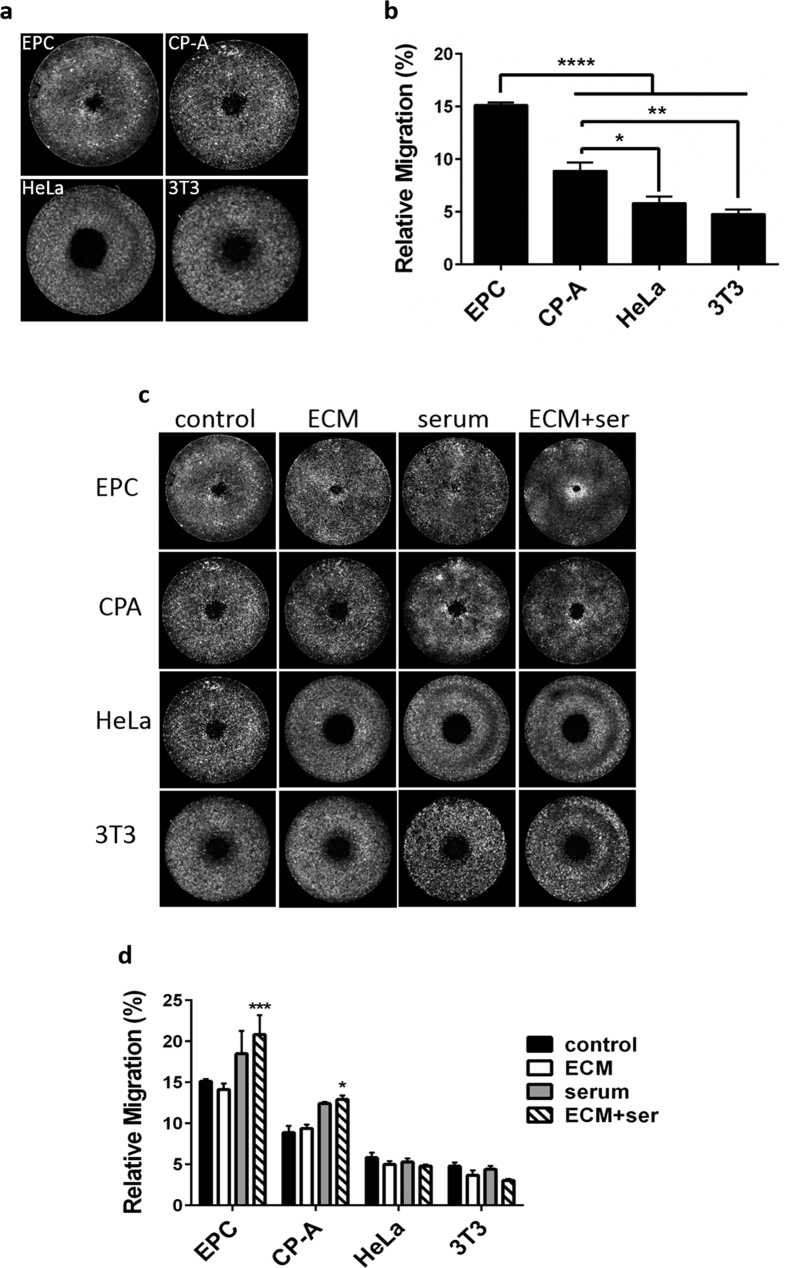
Migration of epithelial and fibroblast cells in unstimulated and stimulated conditions. (**a**) CellTrace migration assays performed as described on EPC2 (EPC), CP-A, HeLa, and Swiss 3T3 (3T3) cells. Migration period was 14 hours. (**b**) Migration results pooled from three independent experiments as in (**a**). Differences in migration were detectible between all cell lines with the exception of HeLa versus Swiss 3T3. (**c**) Cells were allowed to migrate under control conditions, on a collagen coated surface (ECM), with serum stimulation (serum), or with both collagen-coating and serum stimulation (ECM + ser). (**d**) Migration results pooled from three experiments as in (**c**). The combined treatment (ECM + ser) increased migration relative to control in EPC2 and CP-A cells. Graphs in (**b,d**) present the means of three independent experiments and error bars indicate SEM. * indicates p < 0.05, ** indicates p < 0.01, *** indicates p < 0.001, **** indicates p < 0.0001. Data were analyzed by one-way ANOVA with Tukey’s multiple comparisons test.

The CellTrace dyes have been demonstrated to distribute consistently through cell divisions such that cells of each generation contain a 2-fold reduction in dye relative to the previous generation^17^,^18^. In an exclusion zone type migration assay, this property allows evaluation of migration in terms of redistribution of the parental cell population without ambiguity due to proliferation. We evaluated the utility of this approach to distinguish migration rates of various cell lines. We tested, EPC2, CP-A, HeLa, and Swiss 3T3 murine fibroblasts. We were able to easily discern differences in unstimulated migration between all cell lines except the two least motile, HeLa and Swiss 3T3 (Fig. 4a,b). We next evaluated the effects of stimulating cell motility with growth factors or an extracellular matrix (ECM) protein. For growth factor stimulation, cells were serum and growth factor-starved for 12 hours, then allowed to migrate in serum-containing medium. Alternatively, cells were allowed to migrate on a collagen-coated substrate, with or without the serum starvation/stimulation treatment. Migration of EPC2 and CP-A cells was not strongly impacted by either treatment alone, but did significantly increase as a result of the combined treatment. The less motile HeLa and fibroblast cells were unaffected by the treatments (Fig. 4c,d).

**Figure 5:**
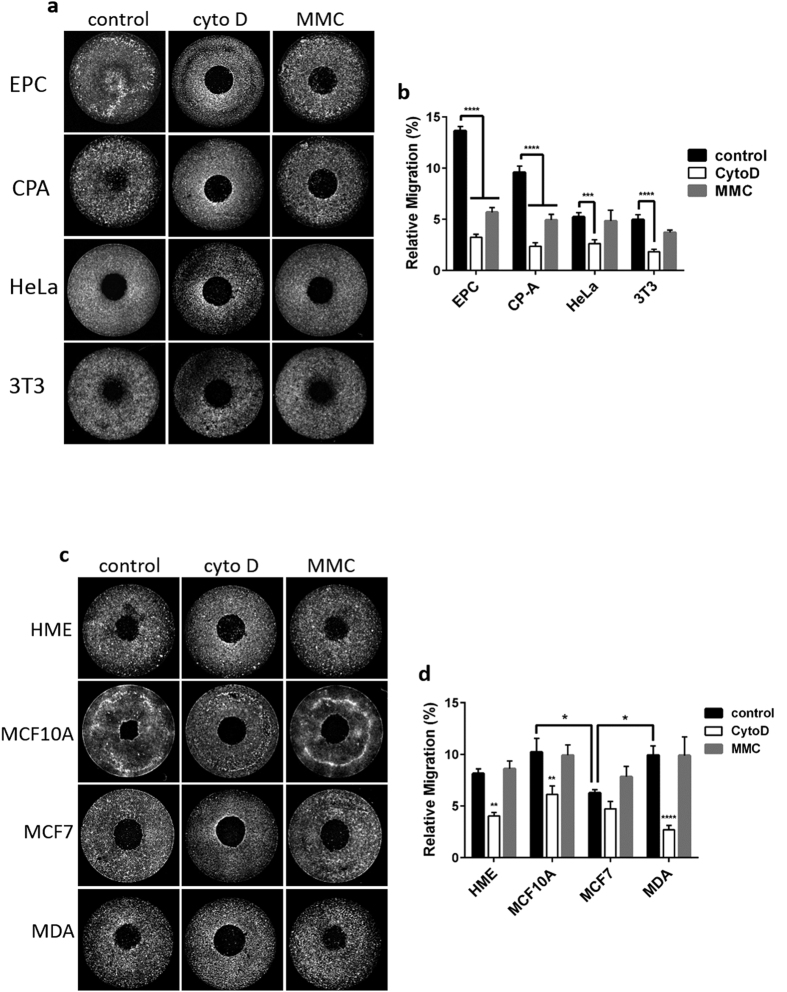
Cell migration is inhibited by cytochalasin D and mitomycin C. (**a**) 14 hour migration assays of EPC2 (EPC), CP-A (CPA), HeLa, and Swiss 3T3 (3T3) cells that were exposed to 1 μg/mL cytochalasin D (cyto D) during migration, or pretreated for 2 hours with 10 μg/mL mitomycin C (MMC) immediately prior to migration. (**b**) Migration results pooled from three independent experiments as in (**a**). Cytochalasin D significantly inhibited migration in all cell types, while mitomycin C inhibited migration of EPC2 and CP-A cells but not HeLa or Swiss 3T3 cells. (**c**) A panel of human breast epithelial cell lines, HME1 (HME), MCF 10A, MCF7, and MDA-MB-231(MDA) were treated with cytochalasin D (cyto D), or mitomycin C (MMC). (**d**) Migration results pooled from three independent experiments as in (**c**). Under control conditions, MCF7 migration was significantly lower than both MCF 10A and MDA-MB-231. Cytochalasin D treatment inhibited migration in all cell lines except MCF7, and mitomycin C had no effect on any cell line. Data in (**b,d**) present the means of three independent experiments each performed in duplicate and error bars indicate SEM. * indicates p < 0.05, ** indicates p < 0.01, *** indicates p < 0.001, **** indicates p < 0.0001. Data were analyzed by two-way ANOVA with Tukey’s multiple comparisons test.

We next evaluated the efficacy of our approach for detecting pharmacologically induced suppression of cell migration and response to mitotic inhibition. We used cytochalasin D, which promotes actin depolymerization^19^, to inhibit cell migration. We also treated cells with mitomycin C (MMC), a cell cycle antagonist. This inhibitor is commonly used in cell migration assays in an attempt to measure migration without the confounding influence of cell proliferation^13^,^14^. In our standard set of epithelial and fibroblast cell lines, we observed that, as expected, cytochalasin D treatment significantly decreased migration in all cell lines (Fig. 5a,b). Interestingly, we also observed that MMC treatment had a measurable impact on some cell types. Migration was reduced by 56%, and 38% in EPC-2 and CP-A cells, respectively. In contrast, HeLa and Swiss 3T3 cell migration was unaffected by MMC treatment (Fig. 5b). We also tested a panel of breast epithelial cell lines in order to evaluate our approach in the context of a well-studied human cancer model. We compared migration of normal mammary epithelial cells (HME1), non-invasive cells derived from fibrocystic disease tissue, MCF 10A, and the invasive adenocarcinoma lines, MCF7 and MDA-MB-231. These cells were treated with both cytochalasin D and MMC. At the standard concentration of 1 μg/mL, cytochalasin D inhibited migration in all breast cell lines with the exception of MCF7. MMC treatment, in contrast, did not affect motility in these cell types (Fig. 5c,d).

### Primary cell migration in normoxia and hypoxia

**Figure 6:**
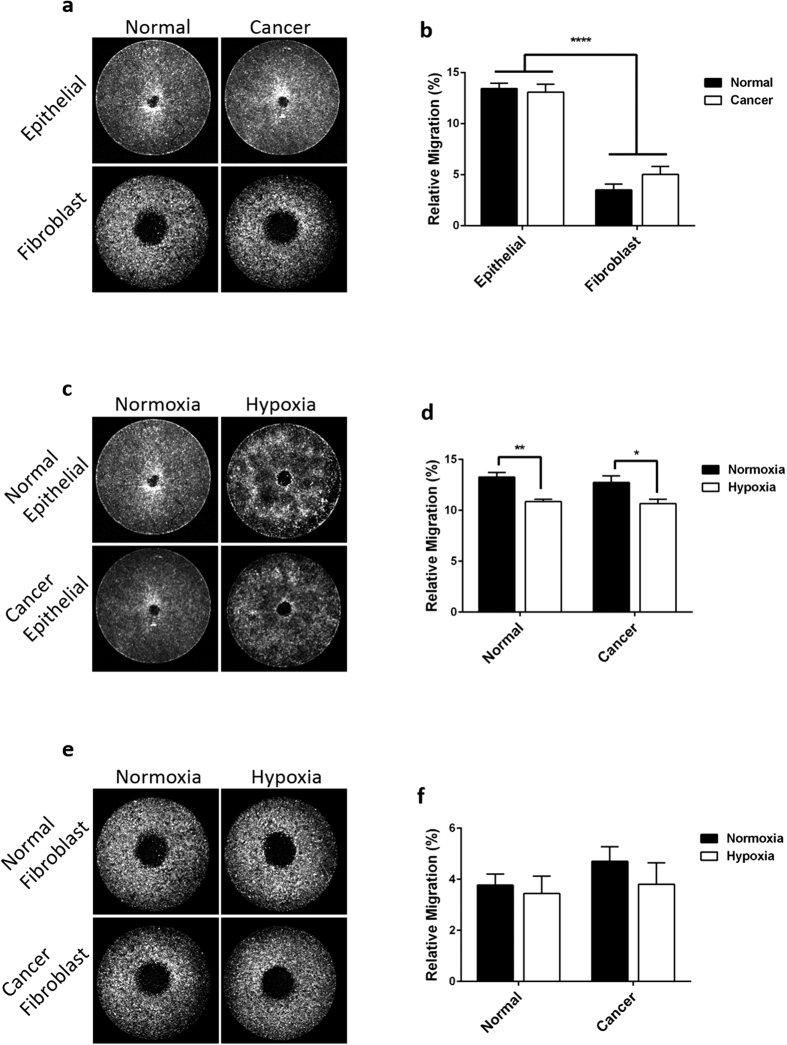
Migration of primary cells from a mouse model of lung cancer. (**a**) Migration of epithelial and fibroblast cells from apparently normal and tumor (Cancer) tissue were isolated from a K-ras G12D mutant mouse lung cancer model. (**b**) Migration results pooled from three independent experiments as in (**a**). Under normal cell culture conditions, epithelial cells were much more migratory than fibroblasts. There was no detectible difference between cells derived from normal tissue and cells from cancer tissue. (**c**) Lung epithelial cells from apparently normal or cancer tissue from a K-ras G12D mutant mouse were exposed to normal cell culture conditions (Normoxia) or hypoxic conditions (Hypoxia, 1% O_2_) during migration. (**d**) Migration results pooled from three independent experiments as in (**c**). Hypoxia significantly reduced migration in both normal and tumor-derived cells. (**e**) Fibroblast cells from a K-ras G12D mutant mouse were exposed to normal cell culture conditions or hypoxic conditions (1% O_2_) during migration. (**f**) Migration results pooled from three independent experiments as in (**e**). Hypoxia had no effect on migration of fibroblasts. Data in (**b,d,f**) are presented as the mean of three independent experiments each performed in 4-fold replicate. Error bars indicate SEM. * indicates p < 0.05, ** indicates p < 0.01, **** indicates p < 0.0001. Data were analyzed by two-way ANOVA with Tukey’s multiple comparisons test.

Finally, we applied this novel migration assay to evaluate primary cells from a mouse model of lung cancer. Lung tissue samples were collected from K-ras G12D mutant mice from either lung nodules (cancer) or normal tissue from the same animal. Epithelial and fibroblast cells were then isolated from both tissue types. We evaluated all four cell types: epithelial and fibroblast cells from normal and cancer tissue, under conditions of normoxia, or hypoxia consisting of 1% O_2_. Under standard, normoxic cell culture conditions, we found that epithelial cells were much more migratory than fibroblasts, but there was no discernible difference between cells derived from tumors and apparently normal tissue (Fig. 6a,b). We found that hypoxic conditions modestly, but significantly reduced migration of both normal and cancer derived epithelial cells (Fig. 6c,d). In contrast, fibroblast cells regardless of tissue origin, were unaffected by oxygen level (Fig. 6e,f).

### Single image modification

**Figure 7:**
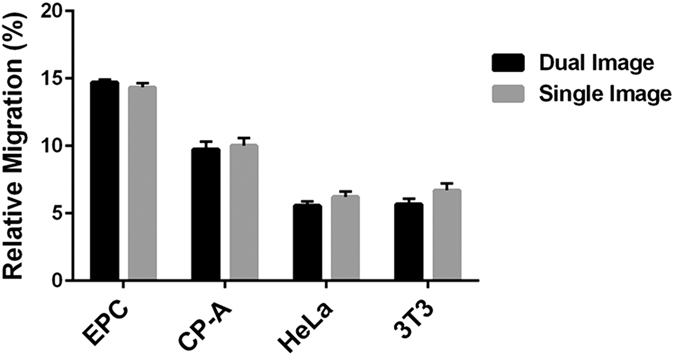
Single image analysis yields similar results to dual image analysis. EPC2 (EPC), CP-A, HeLa, and Swiss 3T3 (3T3) cells were allowed to migrate under normal cell culture conditions. Migration was then evaluated by two methods: 1) the dual image method in which the initial, t_0_ image of each sample was used to define the ROIs that were applied to the the post-migration, t_f_ image (black bars) or 2) the single image method in which a single, standard set of ROIs was applied to all t_f_ images and the pre-migration images were not used in the analysis (gray bars). Results from the two methods were not significantly different (p = 0.2). The two methods provided the same power to distinguish migration of the 4 cell types; all cell lines could be distinguished from each other (p < 0.0001) with the exception for HeLa and Swiss 3T3 cells which were not distinguishable by either method. Data are presented as the mean of 6 independent experiments each performed in duplicate. Error bars indicate SEM. Data were analyzed by two-way ANOVA with Tukey’s multiple comparisons test.

We typically imaged cell distribution at both the start (t_0_) and end (t_f_) of the migration period. The t_0_ image was then used to generate the ROIs that bound the total cell area and the exclusion zone. These ROIs are then applied to the t_f_ image to determine the relative amount of signal that had been transferred to the exclusion zone. This approach effectively controls for any small variation between samples such as imperfect centering of the barriers that form the exclusion zone, or size of the exclusion zone resulting from variation in cell adhesion close to the edge of the barrier. However, these variations appear to be relatively small so we tested whether they might be negligible, thus obviating the need for two rounds of imaging. We first analyzed migration of four cell lines using the dual imaging method. We then generated a single pair of standard ROIs from a t_0_ image of EPC-2 cells, which were not part of the test set. These standard ROIs were then applied to only the t_f_ images of the test set and migration was calculated as usual. Figure 7 compares the results of the two analysis methods. For the cell lines tested, we found no significant difference between the dual image analysis method and the single image method (p = 0.85). More importantly, the two approaches yielded the same statistical power to discriminate the motility of the cell lines. Neither approach showed a significant difference in migration between HeLa and Swiss 3T3 cells; CP-A cells were shown to be significantly more motile than 3T3 cells with a p-value < 0.001; and all other cell lines were differentiated with p < 0.0001 by either analytic method. These results suggest that for most purposes, a single, standard set of ROIs can be applied to multiple samples, dramatically increasing throughput and simplicity of the assay.

## Discussion

We describe here a simple modification to a widely used cell migration assay that disambiguates cell migration from cell proliferation. In traditional migration assays, cells are allowed to migrate into an initially cell-free exclusion zone. At the end of the migration period, cells located in the exclusion zone are simply enumerated. This traditional approach cannot distinguish cells that have physically crawled into the exclusion zone from cells that were “born” there, analogous to estimating national immigration rate by only measuring increases in population. The new strategy presented here circumvents this problem by use of a label that essentially tracks location of the original cell population without regard to proliferation events. By labeling cells with a fluorescent lineage tracing dye at the beginning of the assay, migration can be evaluated in terms of the original or parental cell population. For example, if a cell moves into the exclusion zone during the migration period then undergoes mitosis, the assay reads out the fluorescence of the two daughter cells as the equivalent of the single parental cell and evaluates this scenario as a single migration event. The assay is similarly unaffected by cell divisions that occur prior to migration or in cells that do not migrate into the exclusion zone.

We determined that the CV and CFR dyes, do not significantly impact relevant cellular processes such as migration rate or proliferation. However, it is also critical for meaningful data interpretation that the fluorescent tracking dyes are symmetrically inherited to daughter cells and the dye is stably retained in the cytosol. CV and CFR have been shown to evenly distribute to cell progeny at each division^16^,^20^. Both dyes have also been demonstrated to be well retained in cultures over time and to be minimally transferred between neighboring cells^16^,^17^,^18^,^20^. However, these dye retention studies were done by flow cytometry and may not be directly comparable to our system. Therefore we used image cytometry to confirm stable dye retention in densely cultured, adherent cells and minimal cell-to-cell contamination.

We demonstrated our approach on a wide range of cell types including epithelial cells, fibroblasts, human and mouse cells, cancerous and non-cancerous cells of the same lineage, and both immortalized cell lines and primary cells. Since this assay reports cell migration as a ratio of total cells, it is internally controlled for a wide range of confounding factors including variations in plating density, pre-migration proliferation rate, efficiency of cell attachment, apoptosis, and efficiency of dye uptake. Controlling for these sources of variability not only improves the reproducibility of results, but also allows for reliable comparisons between different cell types, which is not readily accomplished by traditional approaches.

Our new method is relatively high throughput as we have demonstrated it in a 96-well format based on a commercial migration platform. After cell seeding, and excluding migration time, the assay takes approximately 3.5 hours from initiation of migration through calculation of migration values. Using the single image variation of the technique in which a standard set of ROIs is used to analyze multiple post-migration images, the assay labor time is reduced to about 2 hours. For the 12 cell types that we tested, optimal migration time ranged from 11–24 hours. We achieved the reported hands-on time using a fluorescent microscope equipped with a motorized stage, and a multichannel pipette. However, throughput could be dramatically improved by use of an automated liquid handling system and an automated image analysis pipeline. Since the new approach can employ commercially available barrier or exclusion zone type migration kits, the cost is directly comparable to these formats. The only additional cost of reagents is for the CellTrace stain, which we estimate to add about 40 cents per sample. Also, we found that silicon stoppers, such as those used in the Platypus Oris™ 96-well migration kit can be sterilized and reused at least up to five times with no loss of effectiveness.

Another benefit of our novel approach is that it is highly accessible, and is expected to be easily implementable in nearly any lab that is already outfitted for cell culture and fluorescence microscopy. Our analysis pipeline made use of the NIS-Elements software (Nikon, Melville, NY), but does not require any specialized capability, therefore any image analysis software designed for biological applications, many of which are open source, should perform equivalently. We found that undergraduate students were able to efficiently analyze images after about 30 minutes of instruction.

The assay we present can be multiplexed with other, image-based readouts. Most notably, without additional labeling steps, the lineage-tracing indicator dyes provide useful information on cellular proliferation. Since staining intensity corresponds to cell generation, it may be feasible to correlate proliferation events with position and therefore motility. Other, informative analyses employing fluorescent indicators, might include mitochondrial membrane potential, glucose uptake rate, or apoptotic signaling. It should be noted that the CellTrace dyes are fixable and are retained in the cells even after permeabilization, therefore they can be used in conjunction with approaches that require cell fixation such as immunofluorescent labeling.

We validated the new approach using three human epithelial cell lines derived from non-cancerous, pre-cancerous, and metastatic cancer tissues, and a mouse fibroblast line. We were able to unambiguously distinguish these cell lines from each other in terms of cell motility with the exception of the two least motile. We also validated that we could detect the inhibition of cell migration resulting from pharmacological destabilization of filamentous actin. We could also measure stimulation of cell migration by use of an ECM-coated substrate and serum treatment. Significantly, we found that inhibition of cell proliferation by pre-incubation with mitomycin C caused an inhibition of migration in some cell types, but not others. This is an important finding since this drug is routinely used to block proliferation during migration assays in an attempt to achieve a pure migration response^13^. Our results suggest that this practice adds another confounding factor to migration assays and the effect on cells is unpredictable.

Since our approach yields a measure of relative motility, we were able to use it to directly compare different cell lines to each other. We demonstrated how this capability might be used to evaluate migration potential across a panel of related cell lines from different disease states. We compared migration between four widely studied human breast epithelial cell lines, two non-cancerous and two invasive adenocarcinomas. We demonstrated the cancer cell line, MCF7 was significantly less migratory than both MCF 10A and MDA-MB-231 cells. Interestingly, MCF7 cells were resistant to treatment with the actin destabilizing drug, cytochalasin D at the commonly used concentration. This was the only cell line across all panels tested that did not undergo inhibition with this treatment.

We also employed our migration assay to evaluate the effects of hypoxia on motility of primary cells derived from a mouse model of lung cancer. We found that generally the epithelial cells were considerably more migratory than the fibroblast. Interestingly, the behavior of cells isolated from tumors was not different from cells taken from apparently normal tissue from the same animal. There are few studies in which this property is directly compared in matched normal and tumor samples, however cancer cells are generally assumed to have increased motility relative to normal cells. Our results show that this is not invariably true. A possible explanation for our results is that apparently non-cancerous tissue in the K-ras mouse model may have already undergone alterations that affect cell migration by the time that lung tumors have formed. When these primary cells were treated with hypoxia during migration we observed a decrease in cell migration in epithelial cells. This contrasts with other reports that lung cancer derived cell lines can be stimulated to migrate by hypoxia^21^,^22^,^23^. Our results suggest that hypoxia stimulated migration in cancer cells is not a ubiquitous phenomenon. In order to answer these questions definitively however, additional work is needed. It would be valuable to characterize the cell migration patterns in this mouse model more thoroughly by evaluating pre-cancer time points, control animals, and of course, a larger sample size.

In conclusion, we report here a high throughput, flexible, inexpensive, and simple assay that measures cell migration without the confounding influence of cell proliferation. This approach does not require cell cycle blockade or other perturbing manipulation of normal cell physiology. The method would be valuable for screening pharmacological or genetic inhibition of cancer cell migration. It may also be applied to internally controlled correlation studies that relate migratory behavior with protein distribution or physiological readouts. Finally, it should prove valuable in mechanistic studies aimed at elucidating the signaling events that regulate normal and pathological cell migration.

## Methods

### Reagents

Oris™ 96-well migration kits (CMA1.101) were purchased from Platypus Technologies (Madison, WI). CellTrace Far Red (C34572), CellTrace Violet (C34557), and SYTO 24 Green Fluorescent Nucleic Acid Stain (SYTO 24) (S7559) were purchased from ThermoFisher Scientific (Waltham, MA).

### Cell Culture

Cells were purchased from ATCC (Manassas, VA). Immortalized, esophageal, squamous epithelial cells, EPC2 were a generous gift from Dr. Anil Rustgi, University of Pennsylvania. Mouse tissue was a generous gift from Dr. Landon Inge, St. Joseph’s Hospital and Medical Center. Cells were maintained at 37 °C in a humidified incubator with 5% CO_2_ according to ATCC protocols. The cell lines used were: CP-A (CRL-4027), EPC2, HeLa (CCL-2.2), Swiss 3T3 (CCL-92), MDA-MB-231 (HTB-26), HME1 (CRL-4010), MCF 10A (CRL-10317), MCF7 (HTB-22). EPC2 cells were maintained according to the ATCC protocol for CP-A cells. Primary cells were prepared from lung tissue from a K-ras G12D mouse model of lung cancer. To prepare primary cell cultures, fresh tissue samples were washed 3 times with RPMI containing 5 ng/mL amphotericin B, then minced into 1 to 2 mm pieces. Samples were dissociated with collagenase III in Chang medium (Irvine Scientific, Santa Ana, CA, C101) supplemented with Chang medium supplement (Irvine Scientific, C108) at 37 °C for 3 hours with occasional shaking. Cells were pelleted and expended in Chang medium, changed every 3 days. Epithelial and fibroblast cell populations were isolated by multiple rounds of differential trypsinization.

### Migration assays

Cell were seeded into an Oris™ 96-well plate with silicon stoppers in place. Oris™ silicon stoppers were cleaned, sterilized, and re-used up to 5 times will no loss of effectiveness. To reuse, sterile stoppers were firmly inserted into 0.4 mL, 96-well optical bottom plates. Cell density was optimized for each cell type such that after overnight attachment, cells had just reached confluence. Depending on cell type this required 3 × 10^4^–6 × 10^4^ cell per well. After attachment, stoppers were removed according to the manufacturer’s protocol, growth medium was aspirated and cells were labeled with CellTrace for 20 minutes at 37 °C. CellTrace Violet (CV) was used at 20 μM and CellTrace Far Red (CFR) was used at 4 μM in Hank’s Balanced Salt Solution (HBSS). After staining, cells were returned to fresh growth medium and imaged immediately then returned to the cell culture incubator for the migration period, which ranged from 12–24 hours, depending on cell type. Migration time was optimized for each experimental cell panel such that cells with the highest migration rate did not completely fill the exclusion zone and cells with the slowest migration rate had sufficient numbers of cells in the exclusion zone that they could be reproducibly detected above background, which is the signal at time point t_0_. At the end of the migration period, cells were imaged to collect the final image, t_f_.

Where indicated, cells were treated with 10 μg/mL mitomycin C for 2 hours before stoppers were removed. Where cytochalasin D was used, 1 μg/mL was added to the cell medium at the start of the migration period, immediately after labeling with CellTrace. For serum stimulation experiments, most cells were incubated in serum-free medium for 12 hours prior to initiation of migration, then switched to normal growth medium containing 10% fetal bovine serum (FBS). For EPC2 and CP-A cells, which are adapted to serum-free growth medium, cells were switched to growth factor-free medium for 12 hours before migration. Then migration was stimulated by switching them to normal growth medium additionally supplemented with 2% FBS. For cell migration experiments on collagen, migration wells were coated with a solution of 0.03 mg/mL type I collagen overnight at 37 °C. The solution was then aspirated and wells were rinsed with distilled water and allowed to air dry. Collagen was prepared from a 0.1 M acetic acid stock solution by dilution with PBS.

Conventional, 96-well cell migration assays were also done using the Oris™ migration kit according to the manufacturer’s protocol. After migration, cells were labeled with SYTO 24 for 20 minutes at a concentration of 500 nM in HBSS. The combined effects of migration and proliferation were evaluated by calculating the sum of fluorescent signal detected in the exclusion zone relative to the total signal in the well.

### Microscopy

For migration assays, live cell imaging was done on a Nikon C2 confocal system using a Nikon T*i* automated microscope with NIS-Elements software, and a 4x plan fluor, air lens, with N.A. of 0.13. CV was excited with a 402 nm laser and emission was detected at 428–463 nm, CFR was excited with a 639 nm laser and emission was isolated with a 660 nm long pass filter, SYTO 24 was excited with a 488 nm laser and emission was detected after a 500–550 bandpass filter.

### Image analysis

Analysis was done using the NIS-Elements software. Images taken at the beginning of migration (t_0_) were used to create regions of interest (ROIs) delineating the entire area of the well with labeled cells (ROI-1) and the cell-free exclusion zone (ROI-2). These ROIs were then copied and pasted simultaneously onto the final image (t_f_) taken at the end of the migration period. Keeping their relative positions fixed, the pair of ROIs was positioned on the t_f_ image by aligning ROI-1 with the external edge of labeled field of cells, which coincides with the edge of the well. The summed intensity of both ROIs was then recorded and the pairs of values were exported to an Excel spreadsheet. Migration was expressed as the percent of signal in the exclusion zone, from ROI-2, relative to the total signal intensity, from ROI-1.

### Proliferation assay

Cells were plated at ~70% confluence in a 96-well plate and exposed to CellTrace dyes at the indicated concentrations. After 24 hours of incubation, cultures were rinsed and labeled for 20 minutes with SYTO 24 at a concentration of 500 nM in culture medium. Cells were rinsed with HBSS and immediately imaged by confocal with excitation at 488 nm and emission at 520 nm. Images of entire wells were thresholded and stained nuclei were automatically counted using NIS-Elements software.

### Dye retention assays

Cells were seeded into a 96-well plate, close to confluent density and labeled with CV or CFR. Cells were imaged at the indicated time points by confocal microscopy using a 20× plan fluor, air lens, with N.A. of 0.75. The entire well was scanned and individual images were stitched to generate a large composite image. NIS-Elements software was used to measure the total fluorescence intensity for the entire well.

### Dye transfer assays

Cells were labeled with CV or CFR in suspension, then seeded either as single-labeled or mixed cultures. After 24 hours, all samples were imaged in both CV and CFR channels as described above. Images were analyzed with CellProfiler cell image analysis software (Broad Institute). Briefly, dye transfer was calculated as the intensity of transferred dye signal in the mixed sample as a percentage of that signal observed in the single labeled samples. See Supplementary information, Supplementary results, and Figure S2 for detailed image analysis protocol.

### Statistical analysis

Data were analyzed in GraphPad Prism version 6.05 (GraphPad, La Jolla, CA) using ANOVA with Tukey’s or Dunnett’s post hoc test for multiple comparisons. Results were considered significant if p < 0.05.

## Additional Information

**How to cite this article**: Glenn, H. L. *et al*. A simple non-perturbing cell migration assay insensitive to proliferation effects. *Sci. Rep.* **6**, 31694; doi: 10.1038/srep31694 (2016).

## Supplementary Material

Supplementary Information
