# Supplementary material for: A simple non-perturbing cell migration assay insensitive to proliferation effects
Source: Sci Rep. 2016 Aug 18;6:31694. doi: 10.1038/srep31694 (PMC4989229; doi:10.1038/srep31694)
Supplement: Supplementary Information [file srep31694-s1.pdf]

A simple non-perturbing cell migration assay insensitive to proliferation effects

Honor L. Glenn, Jacob Messner, and Deirdre R. Meldrum

Supplementary Information

Supplementary Results

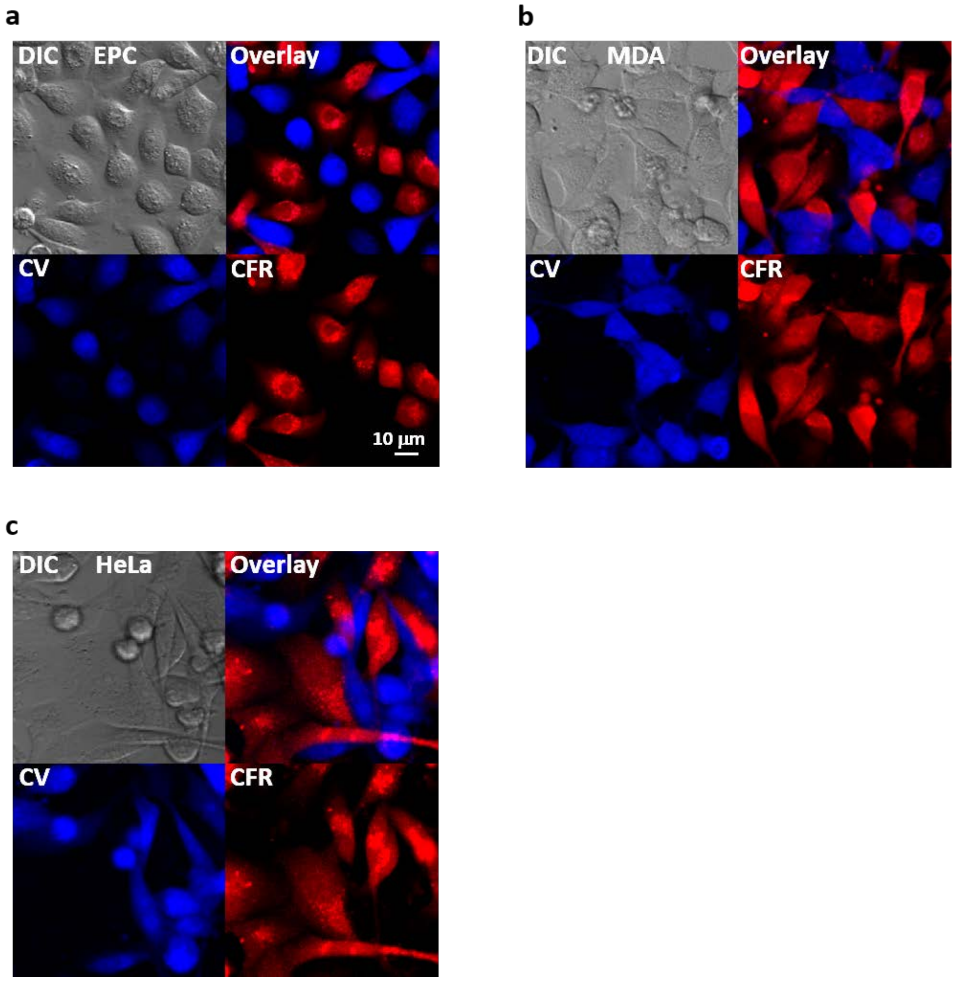

Figure S1. CellTrace cell-to-cell dye transfer. (a) EPC2 cells (EPC), (b) MDA-MB-231 cells (MDA), or (c) Hela cells were labeled with CellTrace Violet (CV) or CellTrace Far Red (CFR) in suspension, then cells were mixed and seeded. After 24 hours, nearly confluent cultures were imaged with confocal microscopy in three channels: differential interference contrast (DIC), laser/emission = 402/446 (CV) or 639/660 long-pass (CFR).

Supplementary methods

Dye transfer image analysis. Images of CV-labeled, CFR- labeled, and mixed cultures of cells were imported into CellProfiler (version 2.1.1). Images of CV (blue) and CFR (red) cells cultured in isolation provided controls for background levels of red fluorescence observed in CV labeled cells, and blue fluorescence in CFR labeled cells. If dye exchange is minimal, it is expected that CV and CFR labeled cells in mixed culture would exhibit similar red and blue

background respectively as their single dyed analogs. Red and blue cells were segmented by enhancing the edges of the cells and thresholding their respective fluorescent images on pixel intensity. The cellular regions were then eroded to circular sampling regions 2.5 microns in diameter to minimize the instances of sampling from cells that physically overlap (Figure S2). Mean intensity values of both red and blue fluorescence were extracted from all sampling regions. The amount of dye exchanged is expressed as the increase in the percentage of foreign fluorescence relative to the percent of native fluorescence determined from cells in single dye cultures (“CV only” or “CFR only”). In all cases, the background fluorescence or fluorescence due to dye transfer was scaled to the total average fluorescence of the cell, the sum of red and blue signal. This was done to account for variation do to geometry such cell thickness or the relative height of the imaging plane through the cell body.

Calculations were done as follows:

$$\text{CFR transfer} = \frac{(\%\ red\ in\ CV\ cells\ in\ mixed\ culture) - (\%\ red\ in\ CV\ cells\ in\ CV\ only\ culture)}{\%\ red\ in\ CFR\ cells\ in\ CFR\ only\ culture}$$

$$\text{CV transfer} = \frac{(\%\ blue\ in\ CFR\ cells\ in\ mixed\ culture) - (\%\ blue\ in\ CFR\ cells\ in\ CFR\ only\ culture)}{\%\ blue\ in\ CV\ cells\ in\ CV\ only\ culture}$$

Where:

$\%\ red\ in\ CV\ cells\ in\ mixed\ culture =$

$$\frac{mean\ red\ fluorescence\ of\ CV\ cells\ in\ mixed\ culture}{mean\ red\ fluorescence\ of\ CV\ cells\ in\ mixed\ culture + mean\ blue\ fluorescence\ of\ CV\ cells\ in\ mixed\ culture} *100$$

$\%\ red\ in\ CV\ cells\ in\ CV\ only\ culture =$

$$\frac{mean\ red\ fluorescence\ of\ CV\ cells\ in\ CV\ only\ culture}{mean\ red\ fluorescence\ of\ CV\ cells\ in\ CV\ only\ culture + mean\ blue\ fluorescence\ of\ CV\ cells\ in\ CV\ only\ culture} *100$$

$\%\ red\ in\ CFR\ cells\ in\ CFR\ only\ culture =$

$$\frac{mean\ red\ fluorescence\ of\ CFR\ cells\ in\ CFR\ only\ culture}{mean\ red\ fluorescence\ of\ CFR\ cells\ in\ CFR\ only\ culture + mean\ blue\ fluorescence\ of\ CFR\ cells\ in\ CFR\ only\ culture} *100$$

$\%\ blue\ in\ CFR\ cells\ in\ mixed\ culture =$

$$\frac{mean\ blue\ fluorescence\ of\ CFR\ cells\ in\ mixed\ culture}{mean\ blue\ fluorescence\ of\ CFR\ cells\ in\ mixed\ culture + mean\ red\ fluorescence\ of\ CFR\ cells\ in\ mixed\ culture} *100$$

$\%\ blue\ in\ CFR\ cells\ in\ CFR\ only\ culture =$

$$\frac{mean\ blue\ fluorescence\ of\ CFR\ cells\ in\ CFR\ only\ culture}{mean\ red\ fluorescence\ of\ CFR\ cells\ in\ CFR\ only\ culture + mean\ blue\ fluorescence\ of\ CFR\ cells\ in\ CFR\ only\ culture} *100$$

$\%\ blue\ in\ CV\ cells\ in\ CV\ only\ culture =$

$$\frac{mean\ blue\ fluorescence\ of\ CV\ cells\ in\ CV\ only\ culture}{mean\ red\ fluorescence\ of\ CV\ cells\ in\ CV\ only\ culture + mean\ blue\ fluorescence\ of\ CV\ cells\ in\ CV\ only\ culture} * 100$$

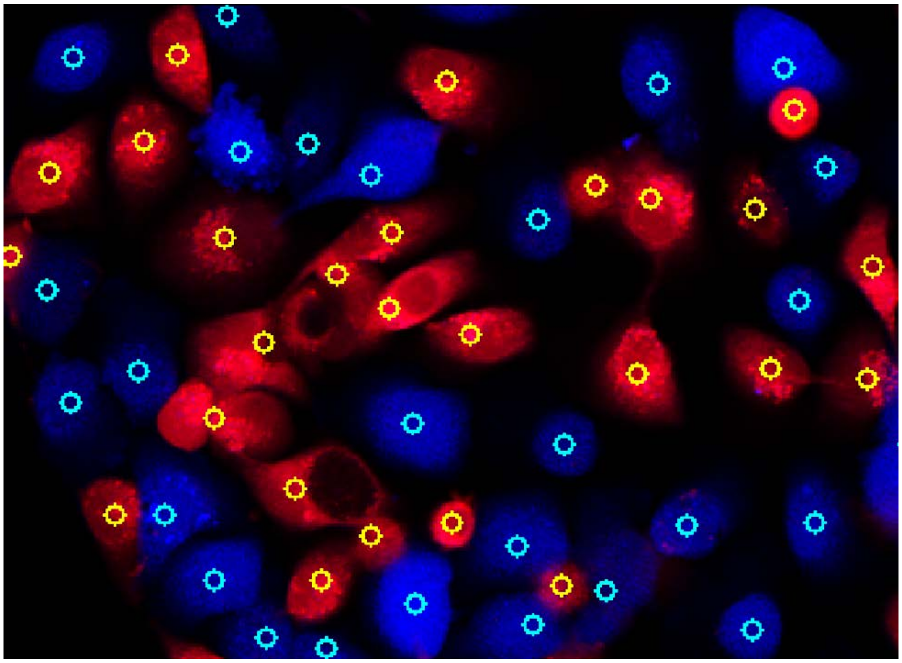

Figure S2. Regions of interest for cell-cell dye transfer analysis. CellProfiler was used to detect predominantly red (CFR-labeled) and predominantly blue (CV-labeled) cells in mixed cultures. The detected cells were then eroded to a central 2.5 micron region of interest indicated in yellow on CFR-labeled cells and cyan on CV-labeled cells.
